# Prevalence of Osteoporosis in Cirrhosis: A Systematic Review and Meta-Analysis

**DOI:** 10.7759/cureus.33721

**Published:** 2023-01-12

**Authors:** Jennifer Kang, Harishankar Gopakumar, Srinivas R Puli

**Affiliations:** 1 Gastroenterology and Hepatology, University of Illinois, Peoria, USA

**Keywords:** osteoporosis, vitamin d deficiency and liver fibrosis, prevalence study, osteoporotic fracture, live cirrhosis

## Abstract

The prevalence of osteoporosis in individuals with cirrhosis varies based on the diagnostic approach and etiology of the underlying liver disease. This systematic review aims to evaluate the prevalence of osteoporosis in individuals with cirrhosis. Electronic databases were searched for studies reporting the prevalence of osteoporosis among patients with cirrhosis. The primary outcome was the presence of osteoporosis, as determined by a dual-energy x-ray absorptiometry (DEXA) scan. Secondary outcomes were levels of biochemical markers of bone metabolism, including calcium, vitamin D, phosphorus, and parathormone (PTH) levels. A cohort of 836 patients from 10 studies was included in the final analysis. The pooled rate of osteoporosis was 14.80% (95% CI: 14.19-15.49). Pooled levels of biochemical markers of bone metabolism were as follows: calcium 9.09 mg/dL (95% CI: 8.73-9.45), 25-hydroxyvitamin D (25-OH vitamin D) 15.41 ng/mL (95% CI: 14.79-16.03), phosphorus 15.41 mg/dL (95% CI: 2.99-3.51), and PTH 26.58 pg/mL (95% CI: 25.45-27.71). Pooled levels of liver biochemistries were: bilirubin 3.04 mg/dL (95% CI: 2.84-3.25), aspartate aminotransferase (AST) 65.35 U/L (95% CI: 61.39-69.31), alanine aminotransferase (ALT) 50.17 U/L (95% CI: 46.18-54.10), alkaline phosphatase 133.31 U/L (95% CI: 124.89-141.73), and albumin 3.25 g/dL (95% CI: 3.05-3.45). Cirrhosis appears to be associated with an increased risk for osteoporosis, with a pooled prevalence of 15%. This can include men and individuals younger than 50 years of age, a cohort not typically considered to be at an increased risk of osteoporosis. Levels of 25-hydroxyvitamin D and insulin-like growth factor-1 (IGF-1) were also significantly low. Further studies are required to evaluate the risk of osteoporosis based on the etiology and stage of cirrhosis, especially in younger males, to incorporate this into future prediction models for fragility fractures.

## Introduction and background

There has been a steady increase in the global prevalence of osteoporosis in the past decade [1,2]. Osteoporosis is classified as primary when it is age-related or occurs in postmenopausal women and secondary when caused due to other etiologies [2]. Previous studies have shown that the prevalence of osteoporosis in patients with cirrhosis ranges from 12% to 70% based on the diagnostic approach and etiology of the underlying liver disease [3,4]. The pathophysiology of osteoporosis in cirrhosis is multifactorial and incompletely understood. It is believed to be from the toxic effects and hormonal imbalance resulting from hepatic insufficiency, which leads to an imbalance of osteoblastic and osteoclastic activity [4]. Chronic inflammation resulting from small intestinal bacterial overgrowth and increased flow of bacterial components to the liver has also been proposed to have a role in the pathogenesis of osteoporosis in the individual with cirrhosis [4]. Osteoporosis is characterized by low bone mass, abnormal bone microarchitecture, and increased skeletal fragility. It is diagnosed when the bone mineral density (BMD) is less than 2.5 standard deviations below the peak value obtained from young normal adults and adjusted for gender [4]. BMD is measured using dual-energy x-ray absorptiometry (DEXA) and reported in T-score with a T-score <−2.5 diagnostic of osteoporosis [1]. Risk factors for bone loss include age, estrogen deficiency, alcohol and tobacco use, malnutrition, prolonged steroid use, vitamin D deficiency, and liver cirrhosis [3,5]. Patients with cholestatic liver disease have an increased risk of osteoporosis even without a diagnosis of cirrhosis [3-5]. Previous studies have reported a higher prevalence of osteoporosis in individuals with cirrhosis [4-6] and an increased risk of fracture [7-9]. However, the strength of the evidence is very weak due to the limited number of studies and small sample size. 

It is also essential to recognize that osteoporosis's prevalence alone underestimates the risk of fracture in patients with cirrhosis. As noted by Compston et al. in their review on osteoporosis, the majority of fragility fractures occur in individuals with BMD values above the diagnostic threshold for osteoporosis [10]. Individuals with cirrhosis are at increased risk for fragility fractures, given the intricate manner in which cirrhosis adds to their overall debility [11]. In their meta-analysis of the association between liver cirrhosis and fracture, Liang et al. showed an odds ratio of about two for any fracture risk in patients with liver cirrhosis [8]. Various scoring systems have been proposed to overcome this limitation of T-scores underestimating the risk of fragility fractures. Fracture risk assessment tool (FRAX) [12], Garvan fracture risk calculator [13], and QFracture scores [14] are three independently validated tools used with this intent. It is concerning, however, that chronic liver disease is a component of only one [14] of these three scoring systems, and liver cirrhosis itself is not included in any of them. Most of our current understanding of the pathogenesis and clinical management of osteoporosis in adults comes from data on postmenopausal osteoporosis in women. The true prevalence of osteoporosis in individuals with cirrhosis is unknown, with currently available literature showing contrasting results [11].

Thus, we performed a systematic review and meta-analysis of the current evidence evaluating the association between liver cirrhosis and osteoporosis by reviewing studies assessing the pathophysiology of cirrhosis in its role in osteoporosis [15-24].

## Review

Methods

Search Methodology

We performed a literature review using PubMed, Ovid MEDLINE database, Science Direct, and Google Scholar from inception to September 2021 to identify randomized controlled trials (RCTs), non-randomized prospective and retrospective studies, and case series that reported the prevalence of osteoporosis among patients with cirrhosis. The search terms "cirrhosis," "liver cirrhosis," "osteoporosis," "DEXA," "chronic liver disease," and "bone mineral density" were used in various combinations as part of this literature review. The reference lists of eligible studies were reviewed to identify additional papers, and the retrieved studies were carefully examined to determine and exclude potential duplicate data. Potentially eligible studies were reviewed entirely following selection from the initial search. 

*Study Eligibility* 

Studies were screened for relevance based on the title, abstract, and entire manuscript. To be included in the analysis, studies were required to have enrolled patients with a diagnosis of liver cirrhosis (including viral hepatitis, cholestatic etiologies, autoimmune disorders, alcohol-related, and non-alcoholic fatty liver disease (NAFLD)) who had undergone DEXA and biochemical screening to assess for the prevalence of cirrhosis. Articles were excluded if they were not available in English, if the included patients were under the age of 18, or if they did not have reported outcomes. Each article was reviewed by two investigators independently (JK and HG). Data were extracted from studies meeting both inclusion and exclusion criteria following a review of the entire contents of each paper. Any differences were resolved by a third investigator (SP), discussion, or revision. The agreement was evaluated using Cohen's κ.

*Data Extraction and Quality Assessment* 

The following data were independently abstracted into a standardized form: study characteristics (primary author, year of publication), study design, baseline characteristics of the study population (number of patients enrolled, patient demographics), etiology of cirrhosis, Child-Pugh score, biochemical markers (albumin, aspartate aminotransferase (AST), alanine aminotransferase (ALT), bilirubin, alkaline phosphatase, calcium, phosphorus, 25-hydroxyvitamin D, and parathormone (PTH)), T-score, and Z-score. The risk of bias was rated independently by two authors (JK and HG). 

Outcome Definition

The primary outcome of interest was the presence of osteoporosis. According to the WHO criteria, osteoporosis was defined by a BMD T-score of −2.5 or less and osteopenia by a T-score between −1 and −2.5. The secondary outcomes evaluated were the pooled values of biochemical markers of hepatic and bone metabolism.

Statistical Analysis

This meta-analysis was performed by calculating pooled proportions for outcomes of interest. Individual study proportions were first converted into a quantity using a Freeman-Tukey variant of the arcsine square root transformed proportion. The pooled proportion was calculated as the back-transform of the weighted mean of the transformed proportions, using inverse arcsine variance weights for the fixed effects model and DerSimonian-Laird weights for the random effects model. Forrest plots were created to show the point estimates from each study in relation to the summary pooled estimate. The width of the point estimates in the forest plots indicates the assigned weight to that study. The heterogeneity among the included studies was estimated using the *I*^2 ^test. An *I*^2 ^score of 0%-39% is considered as non-significant heterogeneity, 40%-75% as moderate heterogeneity, and 76% to 100% as considerable heterogeneity. A *p*-value >0.10 rejects the null hypothesis that the studies are heterogeneous. The effect of publication and selection bias on the summary estimates was tested by the Begg-Mazumdar indicator. Funnel plots were constructed to evaluate potential publication bias. Statistical analysis was performed using Microsoft Excel 19 (Microsoft Corporation, New York, USA).

Results

Study Selection and Characteristics

**Figure 1:**
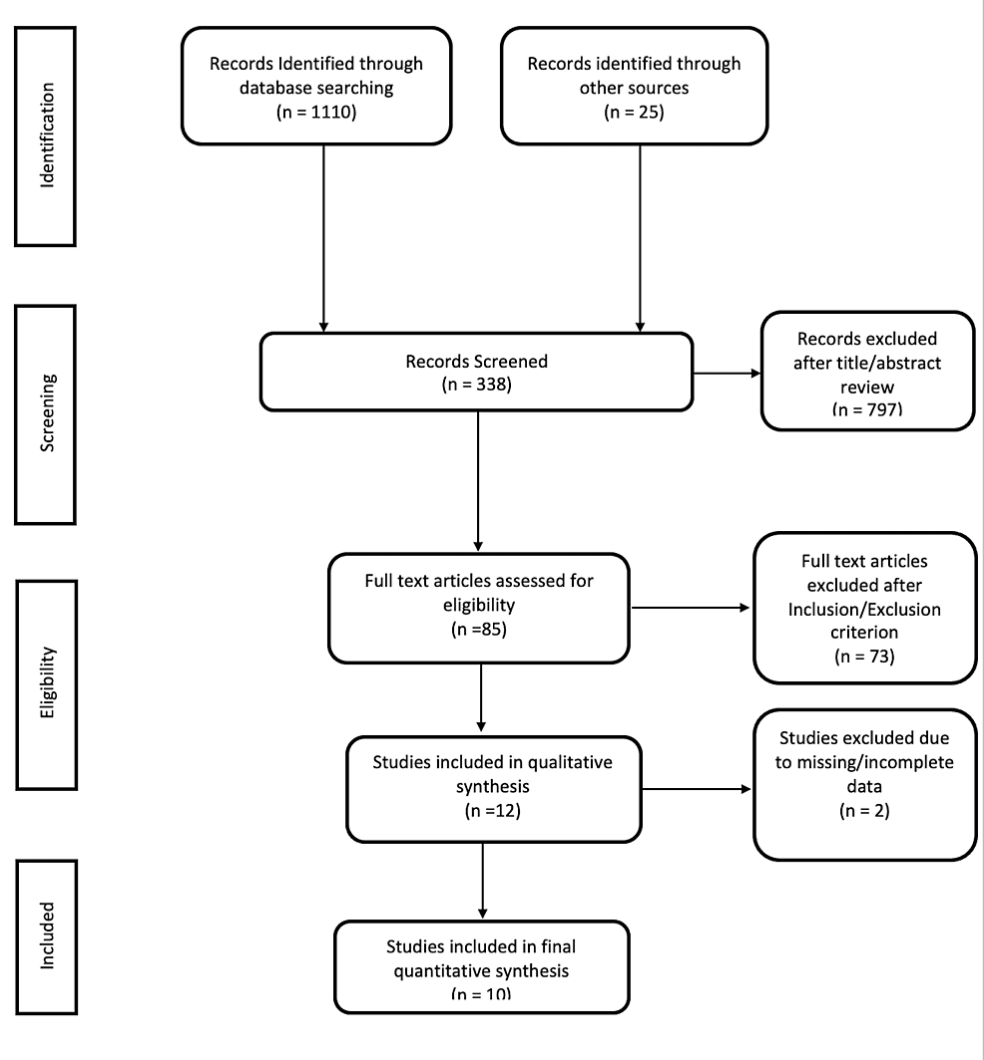
Flow diagram of the search strategy and systematic review.

Our initial search identified 85 articles. Of these, a total of ten studies were included for analysis based on the inclusion and exclusion criteria. All studies were published as full-text articles between 1998 and 2018. A flow diagram of the search strategy and systematic review is shown in Figure 1. The agreement between reviewers was 1.0, as measured by Cohen's κ.

A total of 836 patients were included in this meta-analysis. The mean reported age was 52.12 years ± SD 1.72, and 50.10% of patients were female. The majority of patients had cirrhosis due to alcohol use or a hepatitis C infection. Other etiologies for cirrhosis included hepatitis B, non-alcoholic fatty liver disease (NAFLD), autoimmune, and cryptogenic cirrhosis. The characteristics of the included studies are shown in Table 1.

**Table 1:** Characteristics of studies included in this meta-analysis. PBC: primary biliary cholangitis.

Author, year	Study design	Patients (n)	Mean age (±SD, years)	Males/females	Etiology for cirrhosis
Gonzalez-Calvin et al., 2004 [15]	Case-control	40	59 ± 9.50	100/0	Hepatitis B and hepatitis C
Díez-Ruiz et al., 2010 [16]	Case-control	33	53.09 ± 9.59	100/0	Alcohol
Gallego-Rojo et al., 1998 [17]	Case-control	32	58 ± 9.00	100/0	Hepatitis B and hepatitis C
Mahmoudi et al., 2011 [18]	Case-control	109	60.25 ± 11.2	72/37	Viral hepatitis and alcohol
Mounach et al., 2008 [19]	Case-control	33	47.30 ± 10.40	0/33	PBC
Sokhi et al., 2004 [20]	Single center retrospective	104	54.40 ± 12.90	54/50	Multiple including alcohol and hepatitis C
Zhang et al., 2020 [21]	Case-control	80	51.70 ± 9.60	44/36	Hepatitis B
Carey et al., 2003 [22]	Single center retrospective	207	46.00 ± 6.10	131/76	Hepatitis C and alcohol
González-Calvin et al., 2009 [23]	Case-control	84	65.10 ± 6.25	0/84	Hepatitis C
Muhsen et al., 2018 [24]	Case-control	82	56.50 ± 10	66/34	Multiple including hepatitis B and hepatitis C

**Figure 2:**
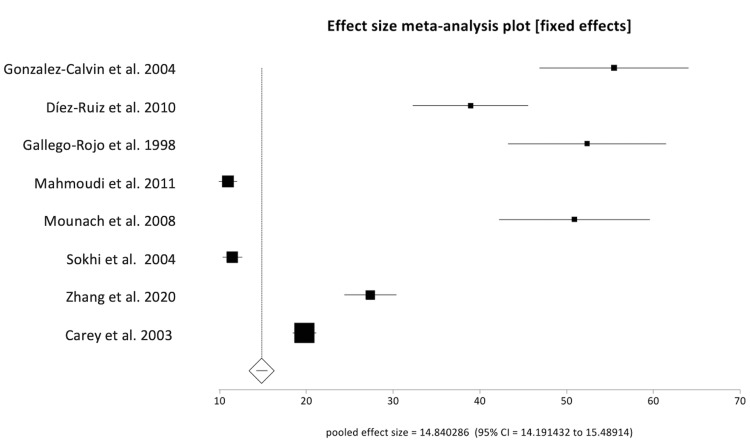
Forest plot showing the individual study rates of osteoporosis in relation to the pooled rate. Source: [15-22]. Note: This image is the author's own creation.

**Figure 3:**
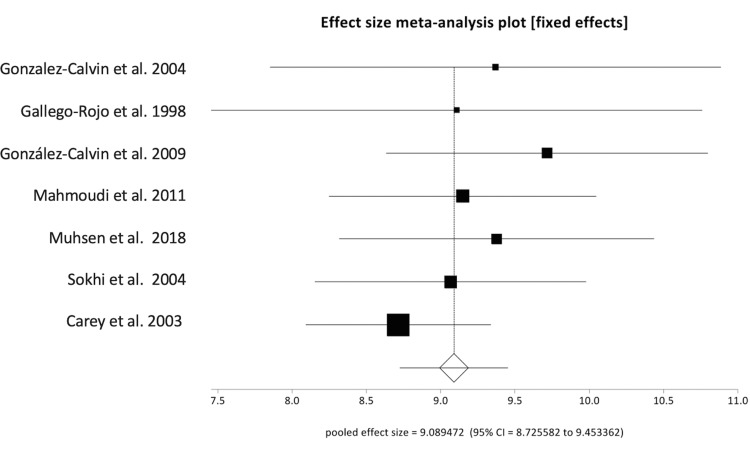
Forest plot showing the individual study proportions of calcium (mg/dL) in relation to the pooled level. Source: [15,17,18,20,22-24]. Note: This image is the author's own creation.

**Figure 4:**
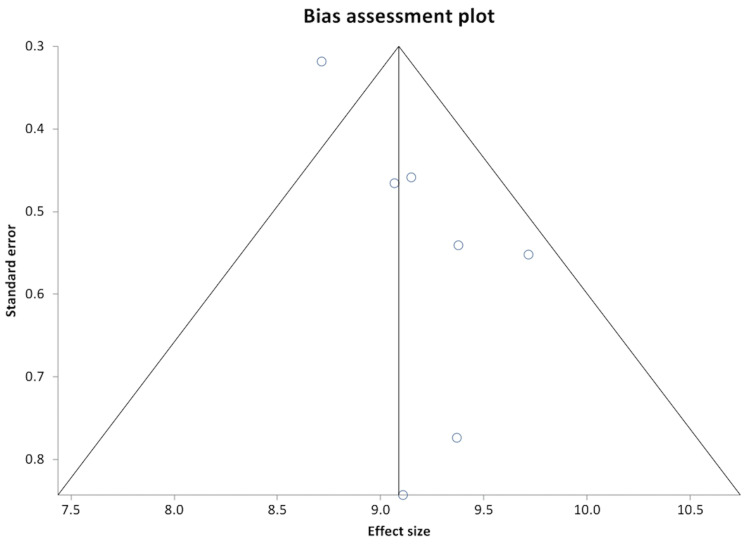
Funnel plot showing publication bias for calcium. Note: This image is the author's own creation.

Reported rates of osteoporosis in included studies ranged from 3% to 28%, with a pooled prevalence rate of 14.84% (95% CI: 14.19-15.49). The Begg-Mazumdar bias indicator gave Kendall's tau-b value of 0.5 (p-value = 0.11), suggesting no publication bias. Forest plot demonstrating the pooled rate of osteoporosis is shown in Figure 2. Pooled values of biochemical markers of bone metabolism were as follows: calcium 9.09 mg/dL (95% CI: 8.73-9.45), 25-hydroxyvitamin D (25-OH vitamin D) 15.41 ng/mL (95% CI: 14.79-16.03), phosphorus 15.41 mg/dL (95% CI: 2.99-3.51), and PTH 26.58 pg/mL (95% CI: 25.45-27.71). Pooled values of liver biochemistries were: bilirubin 3.04 mg/dL (95% CI: 2.84-3.25), AST 65.35 U/L (95% CI: 61.39-69.31), ALT 50.17 U/L (95% CI: 46.18-54.1), alkaline phosphatase 133.31 U/L (95% CI: 124.89-141.73), and albumin 3.25 g/dL (95% CI: 3.05-3.45). These are shown in Table 2. Forest plot showing individual study results and the pooled calcium level is shown in Figure 3. There was no evidence of heterogeneity with an *I^2^* score of 0% (95% CI: 0%-58.50%). The Begg-Mazumdar bias indicator gave Kendall's tau-b value of 0.33 (*p*-value = 0.38), suggesting no publication bias. The Funnel plot showing publication bias for calcium level is shown in Figure 4. 

**Table 2:** Pooled estimates of biochemical markers of hepatic and bone metabolism. AST: aspartate aminotransferase, ALT: alanine aminotransferase, PTH: parathormone, IGF-1: insulin-like growth factor-1.

Biochemical marker	Pooled estimate	95% CI
Bilirubin	3.04 mg/dL	2.84-3.25
AST	65.35 U/L	61.39-69.31
ALT	50.17 U/L	46.18-54.1
Alkaline phosphatase	133.31 U/L	124.89-141.73
Albumin	3.25 g/dL	3.05-3.45
Calcium	9.09 mg/dL	8.73-9.45
Phosphorus	3.25 mg/dL	2.99-3.51
PTH	26.58 pg/mL	25.45-27.71
25-OH vitamin D	15.41 ng/mL	14.79-16.03
IGF-1	15.84 ng/mL	14.12-17.55

The Z-score compares a person's bone density with that of an average person of the same age and gender. The pooled Z-score at the lumbar spine was −0.28 (95% CI: −0.41 to −0.15), whereas the pooled Z-score at the femoral neck was 0.03 (95% CI: −0.10 to 0.16). 

The T-score compares a person's bone density with that of a healthy 30-year-old of the same gender. Analysis showed a pooled lumbar spine T-score of −0.93 (95% CI: −1.07 to −0.80) and a pooled femoral neck T-score of −0.56 (95% CI: −0.73 to −0.38). 

Discussion

This meta-analysis showing an increased prevalence of osteoporosis in individuals with cirrhosis compared to the general population validates the association between cirrhosis and osteoporosis observed in previous studies. A pooled prevalence rate of 15% is significantly higher compared to the age-adjusted osteoporosis prevalence of 12.6% in adults above 50 in the United States [2]. The prevalence rate in this Centers for Disease Control and Prevention (CDC) report was for adults aged 50 and over, while the mean age of study subjects in our pooled analysis was about 52 years. Advancing age is a well-known risk factor for osteopenia and osteoporosis. The findings of a higher prevalence of osteoporosis in a younger population from this study further emphasize the importance of recognizing the role of cirrhosis in the early development of osteoporosis in this population. This CDC report also noted an osteoporosis prevalence rate of only 4.4% in men [2]. The data from our meta-analysis has an equal representation of gender, with 50% being men, and it shows a pooled prevalence rate of 15%. This suggests that the prevalence of osteoporosis in men with cirrhosis is higher than that in the general population. Gonzalez-Calvin et al. observed that bone mass and bone resorption rates did not differ between postmenopausal women with viral cirrhosis compared to healthy postmenopausal women without cirrhosis [23], while there was evidence of increased osteoporosis in men with viral cirrhosis [15]. Hence, our findings of a high prevalence of osteoporosis in cirrhotic individuals at a younger age group, particularly in men, are of great significance as this is not a common cohort where osteoporosis is suspected or evaluated for. We also found that the pooled T-score and Z-score for the entire cohort were low at −0.28 and −0.56. Although not meeting the definition of osteopenia, this again shows the extent of low BMD in this population. Moreover, low levels of vitamin D, which are associated with low bone formation, were very prevalent in this pooled analysis with a value of 15. We also found significantly reduced levels of IGF-1 (15.84 ng/mL), which can cause osteoblast dysfunction, leading to reduced bone formation. Osteoporosis in chronic liver disease is multifactorial, and various risk factors may determine increased bone resorption or decreased bone formation. There are considerable gaps in our understanding of the mechanisms by which cirrhosis contributes to osteoporosis. Hyperbilirubinemia, steroid use, limited physical activity, malnutrition, and alcohol consumption can all contribute to impaired bone mineralization [16]. Although this could not be formally evaluated in this study, it is an important risk factor to be mindful of in this subset of the population.

The findings of this meta-analysis highlight the importance of recognizing the association of osteoporosis with cirrhosis. Further studies are required to evaluate the risk of osteoporosis based on the etiology and stage of cirrhosis, especially in younger males, to incorporate this into future prediction models for fragility fractures.

While our study shows robust results, it does have a few limitations. First, risk factors for decreased bone mass such as alcohol use, malnutrition, and steroid use are often present in individuals with cirrhosis, and it is unclear whether the increased risk of osteoporosis is secondary to hepatic dysfunction itself or these other factors. The studies utilized in this meta-analysis, furthermore, did not all report the values of interest; while analyses were performed with the available data, it is possible that this may have introduced bias. Another limitation could be the relatively small sample size of the available studies. In addition, it remains unclear how much this increased prevalence of osteoporosis corresponds to an increased risk of fracture. Further studies investigating the risk of fracture in those patients with underlying cirrhosis and osteoporosis should be pursued. 

## Conclusions

Cirrhosis appears to be associated with an increased risk for osteoporosis, with a pooled prevalence of 15%. This can include men and individuals younger than 50 years of age, a cohort not typically considered to be at an increased risk of osteoporosis. Levels of 25-hydroxyvitamin D and IGF-1 were also significantly low. Further studies are required to evaluate the risk of osteoporosis based on the etiology and stage of cirrhosis, especially in younger males, to incorporate this into future prediction models for fragility fractures.
